# Cryoprotectant-dependent preservation of cellular viability and oocyte-secreted factors in slow-frozen feline ovarian tissue

**DOI:** 10.14202/vetworld.2026.1119-1131

**Published:** 2026-03-17

**Authors:** Fueangrat Thatsanabunjong, Saritvich Panyaboriban, Supapit Kanthawat, Promporn Raksaseri, Paweena Thuwanut, Kongkiat Srisuwatanasagul, Sayamon Srisuwatanasagul

**Affiliations:** 1 Graduate Program in Veterinary Biosciences, Faculty of Veterinary Science, Chulalongkorn University, Bangkok 10330, Thailand; 2 Department of Anatomy, Faculty of Veterinary Science, Chulalongkorn University, Bangkok 10330, Thailand; 3 Division of Reproductive Medicine, Department of Obstetrics and Gynecology, Faculty of Medicine, Chulalongkorn University, Bangkok, Thailand

**Keywords:** cryopreservation, Dimethyl sulfoxide, ethylene glycol, feline ovarian tissue, follicular development, oocyte-secreted factors, ovarian tissue preservation, slow-freezing

## Abstract

**Background and Aim:**

Cryopreservation of ovarian tissue is an important technique for preserving the reproductive potential of valuable and endangered species and plays a critical role in establishing genetic resource banks. However, the success of ovarian tissue cryopreservation depends largely on the choice of cryoprotective agent (CPA), which must balance protection against intracellular ice formation with potential cytotoxic effects. In feline ovarian tissue, the dense collagen-rich stromal architecture may further limit CPA diffusion, thereby influencing cryopreservation efficiency. This study aimed to evaluate the effects of two commonly used penetrating CPAs, dimethyl sulfoxide (Me_2_SO) and ethylene glycol (EG), individually and in combination, on cellular apoptosis and the preservation of key oocyte-secreted factors (OSFs), growth differentiation factor-9 (GDF9) and bone morphogenetic protein-15 (BMP15), following slow-freezing cryopreservation of feline ovarian tissue.

**Materials and Methods:**

Ovarian tissues were collected from six healthy domestic cats undergoing routine ovariohys-terectomy. Cortical fragments (approximately 2 × 2 × 3 mm³) were randomly assigned to five groups: Fresh control, Cryo-control (no CPA), 10% Me_2_SO, 10% EG, and a combination of 5% Me_2_SO + 5% EG. Tissues were cryopreserved using a programmable slow-freezing protocol with controlled cooling rates and stored in liquid nitrogen. Post-thaw tissue integrity was evaluated through histomorphological examination, apoptosis detection using the TUNEL assay, and protein expression analysis using Western blotting and immunohistochemistry. Statistical analysis was performed using analysis of variance with Bonferroni post hoc testing (p < 0.05).

**Results:**

All cryopreserved groups exhibited significantly higher apoptosis than fresh tissue. However, tissues preserved with Me_2_SO alone or in combination with EG demonstrated apoptotic indices comparable to those of Fresh controls, whereas EG alone and Cryo-control groups showed significantly higher apoptosis. Western blot analysis revealed that both GDF9 and BMP15 protein levels were significantly reduced after cryopreservation. Nevertheless, GDF9 expression was partially preserved in CPA-treated groups compared with Cryo-control tissue. In contrast, BMP15 expression remained markedly reduced in all cryopreserved groups, indicating high cryosensitivity. Immunohistochemical analysis further showed that the combined Me_2_SO + EG treatment better maintained follicular localization and intensity of GDF9 and BMP15 in primordial and primary follicles.

**Conclusion:**

Me_2_SO-based cryopreservation protocols effectively reduce apoptosis and maintain structural integrity in feline ovarian tissue. However, significant depletion of critical OSFs, particularly BMP15, occurs despite preserved morphology. These findings highlight a molecular–structural discrepancy in cryopreserved ovarian tissue and emphasize the need to optimize cryopreservation strategies that preserve both cellular viability and functional molecular signaling essential for successful follicular development and fertility restoration.

## INTRODUCTION

Cryopreservation of ovarian tissue is an important strategy for preserving the reproductive potential of valuable and endangered species and serves as a foundation for establishing genetic resource banks. Domestic cats (*Felis catus*) are widely recognized as valuable models for developing assisted reproductive technologies applicable to threatened wild felids. However, the clinical application of ovarian tissue cryopreservation remains limited by cryoinjury, which is primarily caused by intracellular ice-crystal formation and the cytotoxic effects of cryoprotective agents (CPAs) [1, 2]. Therefore, optimizing cryopreservation protocols, particularly the selection of appropriate CPAs, is essential for improving post-thaw tissue viability and functional integrity [2].

The slow-freezing method is commonly used for ovarian tissue preservation because primordial follicles are more resistant to cryoinjury than mature oocytes [3]. This method relies on CPAs to promote cellular dehydration and reduce intracellular ice formation. Despite their protective role, CPAs may also exert cytotoxic effects, creating a delicate balance between cryoprotection and cellular damage [4]. Consequently, the optimal CPA formulation that maximizes protection while minimizing toxicity in feline ovarian tissue has not yet been fully determined.

The feline ovarian stroma consists predominantly of a dense network of type I collagen fibers, which confers structural rigidity to the tissue and creates a physical barrier that can impede CPA diffusion [5]. This compact stromal architecture restricts the passive penetration of molecules such as dimethyl sulfoxide (Me_2_SO) and ethylene glycol (EG), unlike the comparatively loose and vascularized ovarian stroma observed in murine models [5, 6]. As a result, carefully optimized equilibration procedures are required to ensure that CPAs reach follicles located deep within the cortical tissue while avoiding prolonged exposure that may induce chemical cytotoxicity in superficial tissue layers.

Although previous studies have reported successful structural preservation of feline ovarian tissue with relatively high post-thaw follicular survival rates, the molecular integrity of these tissues remains largely unclear [7]. Traditional evaluation methods primarily rely on histomorphological scoring, which may fail to detect subtle cryoinjury affecting critical oocyte-secreted factors (OSFs). In particular, it remains unclear how slow-freezing protocols influence the preservation of growth differentiation factor 9 (GDF9) and bone morphogenetic protein 15 (BMP15), which are essential regulators of bidirectional communication between oocytes and granulosa cells and play crucial roles in follicular competence [8]. Therefore, the ability of different CPAs to preserve both cellular structure and the molecular signaling environment remains an unresolved issue. This study addresses this knowledge gap by investigating how CPA selection influences cellular apoptosis and the expression of these key developmental proteins, shifting the focus from purely structural evaluation to a functional molecular assessment.

Among the CPAs evaluated in this study, Me_2_SO and EG are widely used penetrating cryoprotectants that replace intracellular water and reduce ice-crystal formation during freezing [9]. However, their effectiveness is limited by cytotoxicity, with EG being particularly associated with mitochondrial dysfunction and DNA damage [10]. In domestic cat ovarian tissue, slow-freezing using 10% Me_2_SO has been shown to preserve preantral follicle ultrastructure more effectively than EG alone or a combination of both CPAs. Transmission electron microscopy analyses have demonstrated fewer ultrastructural abnormalities, such as plasma membrane disruption and detachment between oocytes and granulosa cells, in tissues treated with Me_2_SO, supporting its suitability as a preferred CPA for feline ovarian tissue cryopreservation [7].

These observations highlight the importance of carefully evaluating CPA formulations for reproductive tissue preservation. In addition to structural survival, assessing the functional integrity of cryopreserved tissue is essential for predicting its potential for future applications, such as ovarian tissue transplantation to restore fertility. Oocyte-secreted proteins, including GDF9 and BMP15, serve as key indicators of follicular health and developmental competence. These proteins belong to the transforming growth factor-beta (TGF-β) superfamily and play critical roles in regulating follicular development, granulosa cell proliferation, and overall fertility [11]. Therefore, evaluating the preservation of these OSFs provides important molecular insight into tissue functionality after thawing.

The establishment of functional feline genetic resource banks (GRBs) is a key component of the One Health framework, which promotes biodiversity conservation and sustainable ecosystem health. Long-term ovarian tissue banking represents an important safeguard against extinction for endangered wild felids [12]. However, the true success of such biobanking efforts depends not only on the storage of cryopreserved tissues but also on their readiness for assisted reproductive applications. Ensuring that cryopreserved oocytes retain the molecular signaling mechanisms necessary for follicular activation and development is therefore essential for the successful implementation of ex situ conservation strategies [13].

Despite advances in ovarian tissue cryopreservation, important limitations remain in preserving feline reproductive tissues. Many studies evaluating feline ovarian cryopreservation primarily rely on histomor-phological outcomes, such as follicular and stromal integrity, as indicators of cryopreservation success. Although these assessments provide valuable information regarding structural preservation, they may not accurately reflect the functional competence of follicles after thawing. Cryopreserved tissues that appear morphologically intact may still harbor subtle molecular damage that compromises follicular development and reproductive potential.

A major limitation of current studies is the insufficient understanding of how slow-freezing protocols preserve key OSFs, particularly GDF9 and BMP15, which regulate folliculogenesis and bidirectional communication between oocytes and granulosa cells. These OSFs are essential for maintaining follicular competence, and their disruption may impair follicular activation, growth, and maturation even when structural integrity appears preserved. In addition, the unique structural characteristics of the feline ovary may influence cryopreservation outcomes. The dense collagen-rich cortical stroma of the feline ovary forms a substantial physical barrier that can restrict the diffusion of CPAs into deeper follicular compartments. This structural constraint may alter both the protective efficacy and cytotoxic effects of commonly used CPAs, including Me_2_SO and EG. Although Me_2_SO and EG are widely used penetrating CPAs, their comparative ability to preserve cellular viability and maintain the molecular integrity of OSFs in feline ovarian tissue remains insufficiently understood. Moreover, the potential benefit of combining CPAs to improve tissue penetration while minimizing cytotoxicity has not been fully explored in relation to OSF preservation. Consequently, a significant knowledge gap persists regarding how different CPA formulations influence both apoptosis and the preservation of GDF9 and BMP15 signaling pathways in cryopreserved feline ovarian tissue.

Therefore, this study aimed to systematically evaluate the effects of different penetrating CPAs on the structural and molecular integrity of feline ovarian tissue following slow-freezing cryopreservation. Specifically, the study investigated the comparative effects of Me_2_SO and EG, used individually and in combination, on cellular apoptosis and the expression of the critical OSFs GDF9 and BMP15. By integrating histomorphological evaluation with molecular analyses, including TUNEL assay, Western blotting, and immunohistochemistry, this study sought to determine how CPA selection influences both tissue viability and the preservation of key OSFs involved in follicular development. Ultimately, the findings aim to provide important insights for optimizing feline ovarian tissue cryopreservation protocols, thereby improving the functional preservation of follicles and supporting the development of effective genetic resource banking strategies for domestic and endangered felid species.

## MATERIALS AND METHODS

### Ethical approval

All procedures involving animals were conducted in accordance with internationally accepted guidelines for the ethical use of animals in research. The study complied with the Animal Research: Reporting of *In Vivo* Experiments (ARRIVE) 2.0 guidelines for transparent and responsible reporting of animal-derived data. Ethical approval for the collection and use of feline ovarian tissues was obtained from the Chulalongkorn University Laboratory Animal Center Animal Care and Use Committee under the Animal Care and Use Protocol (CULAC-ACUP; Approval No. 2231035).

Ovarian tissues were collected from clinically healthy domestic cats (Felis catus) undergoing routine elective ovariohysterectomy performed by licensed veterinarians at collaborating veterinary clinics. The surgical procedures were conducted solely for clinical population control purposes and not for research. Only discarded ovarian tissues obtained after surgery were used in this study. Written informed consent was obtained from all animal owners before the surgical procedure, permitting the use of excised tissues for research purposes.

All efforts were made to ensure that no additional harm, stress, or invasive manipulation was imposed on the animals for the purpose of this study. Tissue collection occurred immediately following ovariohysterectomy, and samples were transported to the laboratory under sterile conditions for further processing. Animal welfare standards and biosafety regulations established by Chulalongkorn University were strictly followed throughout the study.

### Study period and location

The study was conducted from June 2024 to December 2025. All experimental procedures, including tissue cryopreservation, histomorphological assessment, and molecular analyses (TUNEL, Western blot, and immunohistochemistry), were performed at the Faculty of Veterinary Science, Chulalongkorn University, Bangkok, Thailand.

### Chemicals and biochemicals

All chemicals used in this study were obtained from several commercial suppliers. Cryopreservation media were prepared using Dulbecco’s Modified Eagle Medium (DMEM) (Cat. No. AL294A; Himedia, Maharashtra, India) and fetal bovine serum (FBS) (Cat. No. A3160401; Gibco, Waltham, MA, USA) as the base media. The CPAs included Me_2_SO (Cat. No. 00124; Loba Chemie, Mumbai, India) and EG (Cat. No. KA-210; Kemaus Chemicals, Cherrybrook, New South Wales, Australia).

For apoptosis detection, the ApopTag® test kit (Cat. No. S-7100; Merck Millipore, Burlington, MA, USA) was used according to the manufacturer’s instructions. For protein analysis, the primary antibodies included rabbit anti-GDF9 polyclonal antibody (Cat. No. AB-93892; Abcam, Waltham, MA, USA), mouse anti-BMP15 polyclonal antibody (Cat. No. SC-271824; Santa Cruz, Dallas, TX, USA), and rabbit anti-β-Actin monoclonal antibody (Cat. No. 4970S; Cell Signaling, Danvers, MA, USA).

For immunoblotting, secondary detection was performed using goat anti-rabbit IgG H&L (HRP) (Cat. No. 7074S; Cell Signaling) and horse anti-mouse IgG H&L (HRP) (Cat. No. 7076S; Cell Signaling). For protein localization, secondary detection was conducted using goat anti-rabbit antibody (PI-10001-1; Vector Laboratories, Burlingame, CA, USA) and horse anti-mouse antibody (BA-2000-1; Vector Laboratories). Tissue sections were processed for immunohistochemistry using the VECTASTAIN® ABC-HRP Kit (Cat. No. PK-4000; Vector Laboratories) and 3,3′-Diaminobenzidine (ImmPACT® DAB) substrate (Cat. No. SK-4100; Vector Laboratories) for signal visualization. All other reagents and instruments used for Western blot analysis were purchased from Bio-Rad (Hercules, CA, USA).

### Animal characteristics and tissue procurement

Ovaries were collected from six (n = 6) healthy domestic cats (*Felis catus*). The donors were mixed breeds, aged between 1 and 3 years, and were in the anestrus phase of their reproductive cycle at the time of surgery. All animals were confirmed to be clinically healthy through pre-surgical examination and had no history of reproductive tract disease or hormonal treatment. Immediately after ovariohysterectomy, the ovaries were placed in sterile saline solution and transported to the laboratory within 3 h for further processing.

### Slow-freezing cryopreservation and thawing protocol

Six feline ovaries were sectioned into cortical fragments (approximately 2 × 2 × 3 mm³) and randomly allocated to five groups: Fresh control and four experimental groups. Four different freezing media were prepared using DMEM supplemented with 10% FBS as the basal medium. The experimental groups included Cryo-control (no CPA) (n = 6), 10% Me_2_SO (n = 6), 10% EG (n = 6), and a combination of 5% Me_2_SO with 5% EG (n = 6).

Tissue fragments were immersed in the respective freezing media at room temperature for 15 min before undergoing a controlled slow-freezing protocol using a programmable temperature controller (Cryologic CL8800i; Cryologic, Mount Waverley, Victoria, Australia). The protocol consisted of cooling at −2°C/min from room temperature to −7°C, manual seeding using cold forceps followed by holding for 10 min, further cooling at −0.3°C/min to −35°C, and finally plunging the samples into liquid nitrogen (−196°C). Samples were stored for a minimum of 24 h to ensure complete thermal equilibrium and cryogenic stability [5].

For thawing, cryovials were first held at room temperature and then placed in a 38°C water bath for 1 min. To remove CPAs, tissue fragments were sequentially washed in PBS containing 50% and 25% of the original CPA concentration, followed by a final wash in 1× PBS (without supplements) for 5 min each [14].

### Histomorphological analysis

The structural integrity of feline ovarian tissue was evaluated using hematoxylin and eosin staining. Paraffin-embedded sections (5 μm thickness) were deparaffinized, rehydrated, and stained to visualize oocytes, granulosa cells (GCs), and the surrounding stromal environment. The stained sections were dehydrated, mounted, and imaged using a Panoramic Digital Scanner (3DHISTECH Ltd., Budapest, Hungary).

Follicle quality was assessed based on established morphological criteria [7]. Briefly, Grade 1 (intact) follicles contained a well-organized granulosa cell layer and an oocyte with a non-pyknotic nucleus and homogeneous cytoplasm. Grade 2 (minor damage) follicles exhibited slight detachment of the oocyte from surrounding GC or mild cytoplasmic vacuolization. Grade 3 (degenerated) follicles showed severe cytoplasmic shrinkage, nuclear pyknosis, or complete detachment from the stromal basement membrane.

A three-grade histological scoring system was applied, where higher scores indicated better follicular integrity (Grade 1 = score 3; Grade 2 = score 2; Grade 3 = score 1). All histological slides were evaluated using a double-blind approach by two independent experienced observers who were unaware of the experimental group assignments.

### TUNEL assay for detecting apoptotic cells

Apoptosis was detected through identification of DNA fragmentation using the ApopTag® Peroxidase In Situ Apoptosis Detection Kit (Merck Millipore, Burlington, MA, USA). Briefly, deparaffinized sections (six sections per group) were subjected to antigen retrieval and endogenous peroxidase blocking. Sections were then incubated with terminal deoxynucleotidyl transferase (TdT) enzyme, followed by incubation with anti-digoxigenin conjugate. Negative controls were prepared by omitting the TdT enzyme, whereas positive controls were treated with DNase I.

The reaction was visualized using ImmPACT® DAB and counterstained with hematoxylin. Apoptotic cells were quantified using the QuantCenter program (3DHISTECH Ltd., Budapest, Hungary) and expressed as the apoptotic index (AI), calculated as [(number of apoptotic cells/total cells) × 100%] across at least 10 microscopic fields with an area of 1 × 1 mm².

### Immunohistochemical analysis

Localization of GDF9 and BMP15 in ovarian follicles was performed using immunohistochemistry as described in our previous study [8]. Briefly, ovarian sections were deparaffinized, rehydrated, and subjected to heat-induced antigen retrieval in citrate buffer (pH 6.0) using a microwave (700 W, two cycles of 5 min each). Endogenous peroxidase activity was blocked using 3% hydrogen peroxide (H_2_O_2_).

Sections were then incubated overnight with primary antibodies against GDF9 and BMP15, followed by incubation with the appropriate secondary antibodies. Immunoreactivity was visualized using ImmPACT® DAB.

Follicles were classified according to developmental stage: primordial follicles (a single layer of flattened GC) and primary follicles (a single layer of cuboidal GC). Protein expression was evaluated based on staining intensity and the proportion of positive cells using the QuantCenter program (3DHISTECH Ltd., Budapest, Hungary), as previously described for other reproductive tissues [15].

Staining intensity was categorized into three levels: weak (1), moderate (2), and strong (3), within a range of 0–300. The H-score was calculated using the formula: H-score = [(0 × % negative cells) + (1 × % weakly positive cells) + (2 × % moderately positive cells) + (3 × % strongly positive cells)]. The expression levels of GDF9 and BMP15 were presented as H-scores for both primordial and primary follicles.

### Western blot analysis (WB)

Immunoblotting was performed in triplicate (n = 6). After thawing, proteins were extracted from ovarian tissues. The tissues were ground in liquid nitrogen and lysed in ice-cold RIPA buffer supplemented with a protease inhibitor cocktail (50 mM Tris-HCl, pH 7.4; 150 mM NaCl; Triton X-100; 0.5% sodium deoxycholate; 0.1% SDS; EDTA).

The supernatant containing 15 µg of protein from each sample was separated by electrophoresis on 8% SDS-PAGE and transferred onto a nitrocellulose membrane. Non-specific binding was blocked using 5% BSA in 1× TBST. Membranes were then incubated with primary antibodies against GDF9 (1:1000), BMP15 (1:200), and β-Actin (1:1000) as a housekeeping protein.

Subsequently, membranes were incubated with HRP-conjugated goat anti-rabbit and horse anti-mouse secondary antibodies diluted at 1:4000. Protein bands were visualized using chemiluminescence and captured using the ChemiDoc Imaging System (Bio-Rad, Hercules, CA, USA). The results were normalized against β-Actin using Image Lab Software (Bio-Rad).

### Statistical analysis

Statistical analysis was conducted using IBM SPSS Statistics (IBM, Chicago, IL, USA). Follicular morphology scores (Grades 1–3, where higher scores indicate better structural integrity) were analyzed using non-parametric statistical methods because the data were ordinal and derived from repeated evaluations of the same samples across different cryopreservation treatments. Overall differences among experimental groups were assessed using the Friedman test. When significant differences were detected, pairwise comparisons between treatments were performed using the Wilcoxon signed-rank test with Bonferroni correction for multiple comparisons.

The percentages of apoptotic cells and protein expression values were expressed as means and analyzed using analysis of variance followed by Bonferroni post hoc tests for multiple comparisons. A p-value < 0.05 was considered statistically significant.

## RESULTS

### Structural preservation across cryopreservation treatments

**Figure 1:**
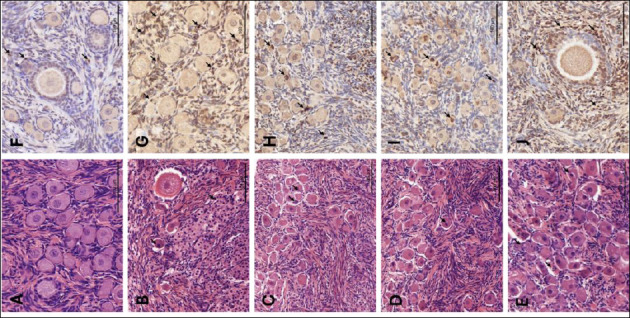
Histological and apoptotic evaluation of feline ovarian tissue. (A–E) Representative images of hematoxylin and eosin (H&E)-stained sections showing morphology of Fresh and cryopreserved groups. (F–J) A, F: Fresh, non-cryopreserved control; B, G: cryopreserved without cryoprotectants (Cryo-control); C, H: 10% Me_2_SO; D, I: 5% Me_2_SO + 5% EG; E, J: 10% EG. Arrows indicate apoptotic bodies. Scale bars = 100 µm. Me_2_SO = dimethyl sulfoxide; EG = ethylene glycol.

Histological examination of the Fresh control group (Figure 1A) revealed a dense ovarian cortex containing numerous healthy primordial and primary follicles. These follicles exhibited clear and well-defined nuclei and maintained a close association with the surrounding stromal cells, indicating high structural preservation. Consistently, the Fresh group exhibited the highest follicular score (2.67 ± 0.49), which was significantly higher than that of all cryopreserved groups (Friedman test, p < 0.001).

In contrast, the Cryo-control group (frozen without CPAs; Figure 1B) exhibited severe cryoinjury. Detachment of oocytes from the granulosa cell layers, extensive cytoplasmic vacuolization, and shrunken pyknotic nuclei within follicles were observed in the Cryo-control group. In addition, the surrounding stroma appeared less organized compared with that of the fresh tissue. Accordingly, the Cryo-control group had the lowest mean morphological score (1.33 ± 0.49), which was significantly lower than that of all CPA-treated groups (p < 0.05).

### Effect of CPAs on follicular morphology

Tissue fragments preserved with CPAs demonstrated varying degrees of morphological protection. The 10% Me_2_SO (Figure 1C) and 5% Me_2_SO + 5% EG (Figure 1D) groups showed the best structural preservation among the cryopreserved groups, with a higher proportion of Grade 1 follicles resembling those observed in the Fresh control. Although minor interstitial gaps were occasionally observed, the oocyte–granulosa cell complexes remained largely intact. These two groups exhibited comparable morphological scores (10% Me_2_SO: 2.39 ± 0.70; 5% Me_2_SO + 5% EG: 2.06 ± 0.73), with no significant difference between them (p > 0.05), and both scores were significantly higher than that of the Cryo-control group (p < 0.05).

The EG-only group (Figure 1E) exhibited more pronounced morphological alterations compared with the other CPA-treated groups, including a higher frequency of follicular detachment and cytoplasmic changes. Nevertheless, this group still maintained better structural integrity than the Cryo-control. The follicular score in the 10% EG group (1.72 ± 0.57) was significantly higher than that in the Cryo-control group (p < 0.05), but remained significantly lower than that in the Fresh group (p < 0.05).

### TUNEL assay

**Figure 2:**
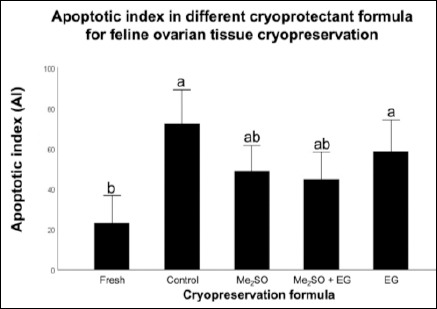
Quantitative analysis of the apoptotic index (AI) across different cryopreservation formulations. The percentages of apoptotic cells were calculated as (number of apoptotic cells / total cells) × 100. Data are expressed as mean ± standard error of the mean (error bars) from n = 6 biological replicates per group. Different lowercase letters (a, b, ab) above bars indicate significant differences (p < 0.05) as determined by one-way analysis of variance followed by Bonferroni post hoc test.

The TUNEL assay successfully identified apoptotic bodies in all experimental groups, which appeared as brown DAB-positive staining (Figures 1F–J). Quantitative analysis revealed a marked increase in the AI in cryopreserved ovarian tissues compared with the Fresh control (23.11 ± 6.82) (Table 1, Figure 2; p = 0.001).

**Table 1:** Effects of different cryoprotectant formulations on apoptotic index and protein expression levels in feline ovarian tissue.

Group	Apoptotic index (%)	GDF9 (relative expression)	BMP15 (relative expression)
Fresh (n = 6)	23.11 ± 6.82^b^	0.87 ± 0.03^a^	0.64 ± 0.08^a^
Cryo-control (n = 6)	72.10 ± 8.50^a^	0.21 ± 0.05^c^	0.15 ± 0.05^b^
Me_2_SO (n = 6)	48.95 ± 6.23^ab^	0.38 ± 0.10^bc^	0.18 ± 0.03^b^
Me_2_SO + EG (n = 6)	44.88 ± 6.68^ab^	0.48 ± 0.10^bc^	0.20 ± 0.04^b^
EG (n = 6)	58.39 ± 8.69^a^	0.59 ± 0.08^ab^	0.22 ± 0.08^b^

Quantitative assessment of cellular apoptosis was determined by the TUNEL assay and oocyte-specific protein expression measured by Western blot across experimental groups. Data are expressed as mean ± standard error of the mean (n = 6 biological replicates per group). Values with different lowercase superscript letters (a, b, c) within the same column indicate statistically significant differences (p < 0.05). Fresh = non-cryopreserved Fresh control, Control = cryopreserved without cryoprotective agents, Me_2_SO = 10% dimethyl sulfoxide, Me_2_SO + EG = 5% dimethyl sulfoxide + 5% ethylene glycol, and EG = 10% ethylene glycol.

Among the cryopreserved groups, none of the cryoprotectant formulations, including 10% Me_2_SO (48.95 ± 6.23), 5% Me_2_SO + 5% EG (44.88 ± 6.68), or 10% EG (58.39 ± 8.69), significantly reduced AI relative to the Cryo-control group (no CPA) (72.10 ± 8.50%) (p = 0.195). However, tissues preserved with Me_2_SO-based formulations showed a noticeable reduction in apoptosis compared with the Cryo-control and EG-only groups, although the differences were not statistically significant.

Notably, only the Me_2_SO-containing groups demonstrated AI values comparable to those of the Fresh control (p = 0.447). In contrast, the EG group maintained a significantly higher AI, which was similar to that observed in the Cryo-control group (p = 0.021).

### WB

**Figure 3:**
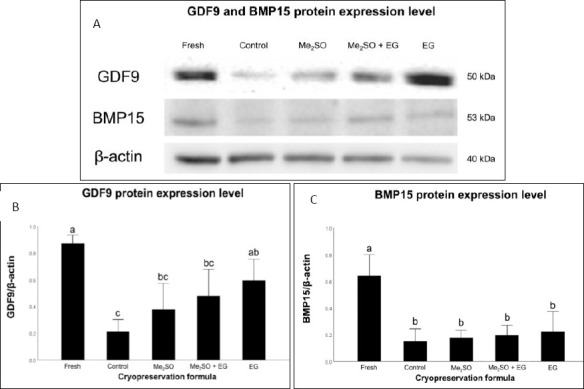
Protein expression of growth differentiation factor 9 (GDF9) and bone morphogenetic protein 15 (BMP15) in cryopreserved feline ovarian tissue. (A) Representative Western blot bands showing GDF9 (~50 kDa), BMP15 (~53 kDa), and β-actin (~42 kDa) as a loading control. (B–C) Densitometric analysis of GDF9 and BMP15 protein expression normalized to β-actin. Fresh = non-cryopreserved Fresh control, Control = cryopreserved without CPAs, Me_2_SO = 10% dimethyl sulfoxide, Me_2_SO + EG = 5% Me_2_SO + 5% ethylene glycol, and EG = 10% ethylene glycol. Bars with different letters indicate statistically significant differences among groups (p < 0.05). Data are presented as mean ± standard error of the mean (n = 6 biological replicates per treatment). Groups with different letters (a, b, c) differ significantly (p < 0.05) according to one-way analysis of variance followed by Bonferroni post hoc analysis.

WB analysis revealed distinct protein bands corresponding to GDF9 and BMP15 at approximately 50 and 53 kDa, respectively (Figure 3). The expression levels of both proteins were highest in Fresh ovarian tissue (0.87 ± 0.03 for GDF9, 0.64 ± 0.08 for BMP15) and significantly decreased across all cryopreserved groups (p < 0.001; Figures 3A and 3B).

For GDF9, the Cryo-control group (0.21 ± 0.05) exhibited the lowest expression. In contrast, tissues treated with CPAs, including Me_2_SO (0.38 ± 0.10, p = 1.00), Me_2_SO + EG (0.48 ± 0.10, p = 0.205), and EG (0.59 ± 0.08, p = 0.015), showed increased expression compared with the Cryo-control group. However, a statistically significant difference was observed only between the Cryo-control and EG groups. Among the CPA-treated groups, the EG group showed the highest GDF9 expression and exceeded the levels observed in the Me_2_SO and Me_2_SO + EG groups, although these differences were not statistically significant.

In contrast, BMP15 expression (Figure 3B) remained significantly reduced and relatively comparable among all cryopreserved groups. No statistical differences were observed between the Cryo-control group (0.15 ± 0.05) and any CPA-treated condition, including Me_2_SO (0.18 ± 0.03, p = 1.00), Me_2_SO + EG (0.20 ± 0.04, p = 1.00), and EG (0.22 ± 0.08, p = 1.00) (Figure 3C).

### Immunohistochemical (IHC) analysis of GDF9 and BMP15

**Figure 4:**
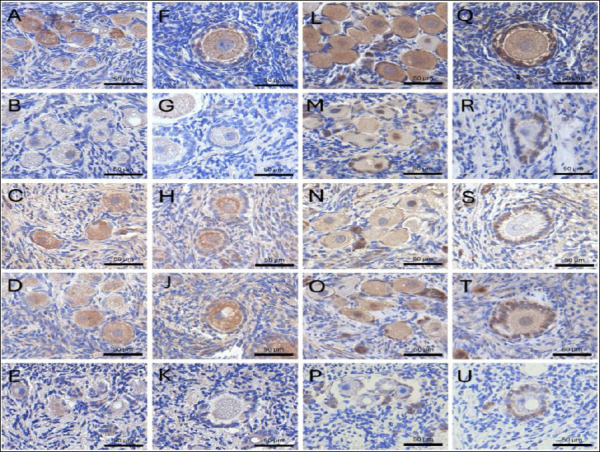
Immunohistochemical localization of growth differentiation factor 9 (GDF9) and bone morphogenetic protein 15 (BMP15) in feline ovarian tissue. Representative images showing the expression of GDF9 (A–K) and BMP15 (L–U) in Fresh and cryopreserved groups. For both proteins, panels represent: (A, F, L, and Q) Fresh, non-cryopreserved control; (B, G, M, and R) cryopreserved without CPAs (Cryo-control); (C, H, N, and S) 10% dimethyl sulfoxide (Me_2_SO); (D, J, O, and T) 5% Me_2_SO + 5% ethylene glycol (EG); and (E, K, P, and U) 10% EG. Staining intensity was predominantly localized within the oocytes of primordial and primary follicles. Scale bars = 100 µm.

IHC analysis of OSFs, including GDF9 and BMP15, provided additional insights into protein localization within the follicular unit. In general, GDF9 and BMP15 localization was detected in the cytoplasm of multiple ovarian cell types, including oocytes, GC, and stromal cells (Figure 4).

**Figure 5:**
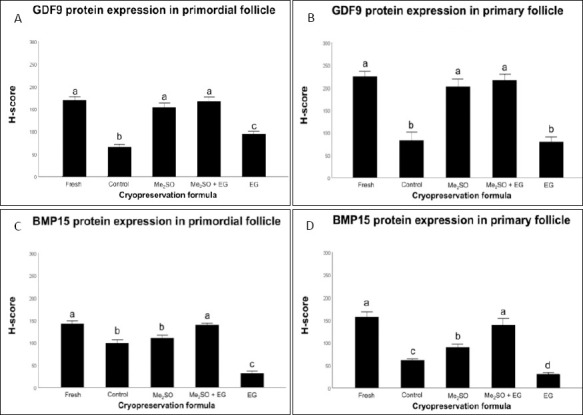
Semi-quantitative H-score analysis of growth differentiation factor 9 (GDF9) and bone morphogenetic protein 15 (BMP15) expression in feline follicles. The bars represent H-score evaluation of oocyte-secreted factors across follicular developmental stages and cryopreservation treatments. (A) GDF9 expression in primordial follicles; (B) GDF9 expression in primary follicles; (C) BMP15 expression in primordial follicles; and (D) BMP15 expression in primary follicles. Values are presented as mean ± standard error of the mean (6 biological replicates per group). Groups labeled with different lowercase letters (a, b, c, d) indicate statistically significant differences (p < 0.05). Me2SO = dimethyl sulfoxide, EG = ethylene glycol.

The results, expressed as H-scores, indicated that the Me_2_SO + EG combination effectively protected the oocyte signaling machinery associated with GDF9 and BMP15 (Table 2). The Me_2_SO + EG group maintained strong GDF9 protein intensity in both primordial (167.06 ± 10.31) and primary follicles (210.97 ± 11.52), which was comparable to the expression levels observed in fresh tissue (170.81 ± 6.27 and 219.32 ± 10.68, respectively) (Figure 5).

**Table 2:** Immunohistochemical H-score analysis of growth differentiation factor 9 (GDF9) and bone morphogenetic protein 15 (BMP15) expression in primordial and primary follicles.

Group	GDF9-positive follicles		BMP15-positive follicles	

	Primordial	Primary	Primordial	Primary
Fresh (n = 6)	170.81 ± 6.27^a^	219.32 ± 10.68^a^	142.84 ± 5.11^a^	157.45 ± 11.12^a^
Cryo-control (n = 6)	65.96 ± 5.64^b^	80.59 ± 17.98^b^	99.82 ± 6.28^b^	62.06 ± 2.59^c^
Me_2_SO (n = 6)	153.80 ± 10.19^a^	196.72 ± 15.86^a^	111.42 ± 5.09^b^	91.10 ± 5.54^b^
Me_2_SO + EG (n = 6)	167.06 ± 10.31^a^	210.97 ± 11.52^a^	139.36 ± 3.56^a^	139.15 ± 14.82^a^
EG (n = 6)	94.98 ± 5.82^c^	77.69 ± 10.00^b^	31.75 ± 5.72^c^	30.61 ± 3.67^d^

Semi-quantitative evaluation of oocyte-secreted factor intensity within specific follicular stages following cryopreservation. Values with different lowercase superscript letters (a, b, c, d) within the same column indicate statistically significant differences (p < 0.05). Fresh = non-cryopreserved Fresh control, Control = cryopreserved without cryoprotective agents, Me_2_SO = 10% dimethyl sulfoxide, Me_2_SO + EG = 5% Me_2_SO + 5% ethylene glycol, and EG = 10% ethylene glycol.

Similarly, the IHC results demonstrated that the dual-CPA formulation maintained BMP15 expression levels in both primordial and primary follicles (139.36 ± 3.56 and 139.15 ± 14.82, respectively) comparable to those observed in Fresh ovarian tissue (142.84 ± 5.11).

Conversely, treatment with EG alone resulted in significantly lower BMP15 expression in primordial follicles (31.75 ± 5.72) and primary follicles (30.61 ± 3.67) compared with the Cryo-control and other CPA-treated groups. In addition, the expression of GDF9 in the Cryo-control group (no CPA; 80.59 ± 17.98) did not differ from that observed in the EG group (77.69 ± 10.00) in primary follicles (p > 0.05).

## DISCUSSION

### Cryopreservation as a fertility preservation strategy in felids

The cryopreservation of feline ovarian tissue is a promising fertility preservation strategy that can be applied to valuable and endangered feline species; however, its success depends on minimizing cellular damage caused by cryoinjury. This study demonstrates that the selection of CPAs profoundly influences both cellular viability and the preservation of crucial functional proteins required for follicular development. While histological integrity has long been considered the gold standard for evaluating cryopreservation outcomes, our findings reveal a significant “molecular–structural” gap in which tissues that appear morphologically intact may still conceal critical injuries at the protein level, particularly within OSF pathways.

### Cellular damage and cryoprotectant-mediated protection

The severe damage observed in the Cryo-control group, characterized by distinct morphological disruption and a high AI of 72.10%, confirmed that intracellular ice formation remains a major obstacle to successful feline tissue cryopreservation [16]. The introduction of penetrating CPAs is essential to mitigate this damage; however, their effectiveness must be balanced against inherent chemical cytotoxicity [17].

In the present study, tissues treated with Me_2_SO, either alone or in combination with EG, maintained apoptotic indices comparable to those of Fresh controls. In contrast, EG alone failed to provide protection comparable to the Cryo-control. This difference in cryoprotectant strategy highlights a species-specific challenge, as the dense and collagenous morphology of the feline ovarian stroma forms a substantial physical barrier to CPA diffusion. This finding suggests that the exposure duration required for a 10% concentration of EG to penetrate deeper feline cortical follicles may exceed the threshold required to maintain mitochondrial and DNA stability in feline ovarian cells, thereby replacing chemical injury with ice-crystal damage.

When the apoptosis results were compared with OSF-related protein findings, a notable inconsistency was observed in the EG-only group. The elevated level of apoptosis (58.39%) accompanied by relatively well-maintained GDF9 protein expression (0.59 ± 0.08) suggests that EG toxicity in the feline ovary may be selective. EG may preferentially preserve oocyte-derived proteins while simultaneously exerting more severe cytotoxic effects on surrounding stromal or GC. This selective impact may position EG as a molecularly permissive but cytologically toxic CPA.

In addition, although combinations of cryoprotectants are generally assumed to provide synergistic benefits, our results demonstrated that the Me_2_SO + EG combination did not outperform Me_2_SO alone in terms of reducing apoptosis or enhancing protein expression. This observation suggests that CPA synergy is species- and endpoint-dependent, particularly at the molecular level. Consequently, our findings challenge the prevailing assumption that CPA cocktails are universally superior for all ovarian tissue types.

### Differential preservation of GDF9 and BMP15

A novel and critical aspect of this study is the evaluation of GDF9 and BMP15 expression, which are key members of the TGF-β superfamily that regulate early folliculogenesis and oocyte–granulosa cell communication [8]. Our WB results indicated differential sensitivity between these two proteins.

Although all CPAs maintained GDF9 expression significantly better than the Cryo-control group, none of the treatments were able to fully prevent a marked decline in BMP15 levels. This observation suggests that BMP15 is a more fragile molecular component of feline ovarian tissue, possibly due to its thermolabile structure or a higher susceptibility to oxidative stress during the freezing–thawing cycle.

From a clinical perspective, compromised BMP15 signaling may threaten the developmental competence of follicles, even when they appear morphologically normal under microscopic examination. Such molecular deficiencies may explain why many cryopreserved follicles fail to reach maturation during subsequent culture or transplantation procedures.

### Stage- and tissue-specific preservation of OSFs revealed by immunohistochemistry

In addition to WB findings, the most significant discovery of this study relates to the stage- and tissue-specific expression patterns of OSFs revealed through IHC analysis. WB analysis provided an overview of protein decline, indicating a generalized reduction of GDF9 and BMP15 within total tissue homogenates, whereas IHC-derived H-scores provided more detailed insights into the follicular microenvironment.

Specifically, the combination of Me_2_SO and EG demonstrated superior protection of the oocyte signaling machinery, maintaining GDF9 and BMP15 expression in primordial and primary follicles at levels statistically comparable to those observed in fresh tissue. These findings suggest that although the dense collagen-rich feline stroma may undergo substantial molecular degradation during slow-freezing, the dual-CPA formulation effectively penetrates the cortical environment and protects critical OSFs within the follicles.

The synergistic action of this dual-CPA strategy is likely associated with the different molecular weights and permeability characteristics of Me_2_SO and EG. By employing both CPAs at lower concentrations (5% each), the protocol may achieve deeper tissue infiltration while remaining below the threshold of chemical cytotoxicity observed in the 10% EG-only group.

Conversely, the pronounced depletion of BMP15 in the 10% EG-only group, where H-scores were significantly lower in primordial follicles, highlights a form of persistent “molecular injury” despite the presence of a cryoprotectant. Considering that GDF9 and BMP15 act as essential regulators of follicle recruitment and prevention of the post-transplantation “burn-out” effect, these IHC findings suggest that combination CPAs are crucial for ensuring that preserved genetic material retains the developmental competence required for successful reproductive conservation programs in Felidae species.

Furthermore, detailed IHC-based molecular profiling may serve as a robust surrogate marker for resource-intensive functional assays such as xenotransplantation or long-term *in vitro* culture in future studies. The primary cause of graft failure following ovarian tissue transplantation is the premature activation or “burn-out” of primordial follicles [8, 18]. Since GDF9 and BMP15 regulate follicular activation dynamics [19], their preservation through optimal CPA selection may serve as a reliable molecular predictor of transplanted tissue functionality and a valuable indicator of tissue quality for future clinical applications [20].

### Limitations and future directions for optimizing feline ovarian cryopreservation

Although the present study establishes a critical association between CPA selection and OSF preservation, several methodological improvements remain necessary for further optimization. A primary limitation of this investigation is the reliance on structural and protein expression endpoints. Although the IHC evaluation of GDF9 and BMP15 may function as a robust molecular surrogate for tissue competence, future studies should incorporate longitudinal functional assays. Techniques such as *in vitro* follicle culture or xenotransplantation into immunodeficient models are essential to determine whether the observed molecular preservation ultimately translates into successful follicular maturation and developmental competence.

Furthermore, the physiological barrier created by the dense collagen-rich feline ovarian cortex may require more sophisticated cryoprotective formulations. Although the Me_2_SO and EG combination demonstrated promising results, future investigations should conduct dose–response studies to identify optimal concentration ratios that maximize cryoprotection while minimizing chemical cytotoxicity. The inclusion of non-penetrating cryoprotectants such as sucrose or trehalose may also warrant investigation, as these agents can facilitate extracellular dehydration and thereby reduce the required concentration of intracellular CPAs.

Finally, elucidating the specific molecular pathways responsible for apoptosis and protein degradation during the freeze–thaw cycle will be crucial. By identifying the mechanical and oxidative stress mechanisms responsible for BMP15 loss, targeted antioxidant or anti-apoptotic supplementation strategies could be incorporated into existing protocols. Such advancements will be essential for transforming feline GRBs from passive storage systems into functional platforms capable of restoring the reproductive potential of threatened species.

## CONCLUSION

This study demonstrates that the selection of CPAs plays a critical role in determining both the structural integrity and molecular functionality of cryopreserved feline ovarian tissue. Histomorphological evaluation showed that tissues preserved with Me_2_SO -based formulations, particularly 10% Me_2_SO and the combination of Me_2_SO + EG, maintained significantly higher follicular integrity compared with the Cryo-control group. In contrast, tissues frozen without CPAs exhibited severe cryoinjury, including follicular detachment, cytoplasmic degeneration, and a markedly elevated AI. Although EG alone provided some structural protection relative to the Cryo-control, it resulted in higher levels of apoptosis compared with Me_2_SO-containing treatments.

At the molecular level, WB analysis revealed that cryopreservation significantly reduced the expression of OSFs, particularly GDF9 and BMP15, compared with Fresh ovarian tissue. While CPA treatments partially preserved GDF9 expression, BMP15 was markedly more sensitive to the freezing–thawing process and declined substantially across all cryopreserved groups. However, IHC analysis demonstrated that the dual-CPA formulation (Me_2_SO + EG) effectively maintained the spatial expression of GDF9 and BMP15 within primordial and primary follicles at levels comparable to fresh tissue. These findings indicate that although overall protein levels may decline during cryopreservation, targeted CPA combinations can still preserve critical oocyte signaling pathways within the follicular microenvironment.

From a practical perspective, these findings highlight the importance of integrating molecular biomarkers, such as GDF9 and BMP15, with traditional histological evaluation to accurately assess the functional competence of cryopreserved ovarian tissue. Reliance solely on morphological criteria may overlook subtle molecular injuries that compromise follicular development and long-term fertility potential. Therefore, evaluating OSFs provides a more reliable indicator of tissue quality and may serve as a predictive marker for the success of subsequent applications, including ovarian tissue transplantation or *in vitro* follicle culture.

A major strength of this study lies in the combined use of histomorphological assessment, apoptosis analysis, WB, and IHC to provide a comprehensive evaluation of cryopreservation outcomes. This integrative approach allowed the identification of a “molecular–structural” discrepancy, revealing that tissues appearing morphologically preserved may still experience significant molecular damage. The study also provides new insights into the species-specific challenges associated with the dense collagen-rich stroma of the feline ovary, which can restrict CPA diffusion and influence cryoprotective efficiency.

In conclusion, the findings indicate that Me_2_SO-based cryopreservation strategies, particularly when combined with EG at lower concentrations, provide improved preservation of both structural integrity and key OSFs in feline ovarian tissue. The preservation of GDF9 and BMP15 signaling pathways may serve as a crucial determinant of follicular developmental competence following cryopreservation. These results contribute valuable knowledge for optimizing ovarian tissue banking protocols and support the development of functional GRBs for endangered felids, thereby advancing assisted reproductive technologies and biodiversity conservation within the One Health framework.

## DATA AVAILABILITY

The data that support the findings of this study, including raw quantitative datasets, original uncropped immunoblot images, and histopathological micrographs, are available from the corresponding author upon reasonable request.

## GENERATIVE AI USAGE STATEMENT

During the preparation of this work, the authors used Gemini 2.5 to scrutinize the language for grammar, spelling, and style, as well as to improve clarity. After using this tool, the authors reviewed and edited the content as needed and took full responsibility for the content of the publication.

## AUTHORS’ CONTRIBUTIONS

FT: Conceptualization, methodology, validation, formal analysis, investigation, writing – original draft, writing – review & editing, visualization. SP: Conceptualization, methodology, validation, formal analysis, investigation, writing – review & editing, supervision. SK: Methodology, validation, formal analysis, investigation. PR, PT, and KS: Methodology, validation, formal analysis, investigation, supervision. SS: Conceptualization, methodology, validation, formal analysis, investigation, writing – original draft, writing – review & editing, visualization, supervision, funding acquisition.
